# Association of dietary nutrient intake with type 2 diabetes: A Mendelian randomization study

**DOI:** 10.1097/MD.0000000000038090

**Published:** 2024-05-10

**Authors:** Ruixiang Kang, Dong Guo, Jiawei Wang, Zhencong Xie

**Affiliations:** a Shandong University of Traditional Chinese Medicine, Jinan, China.

**Keywords:** amino acids, Mendelian randomization, nutrients, two samples, type 2 diabetes

## Abstract

Observational research suggests that the evidence linking dietary nutrient intake (encompassing minerals, vitamins, amino acids, and unsaturated fatty acids) to type 2 diabetes (T2D) is both inconsistent and limited. This study aims to explore the potential causal relationship between dietary nutrients and T2D. Causal estimation utilized Mendelian randomization techniques. Single nucleotide polymorphisms linked to dietary nutrients were identified from existing genome-wide association studies and used as instrumental variables. Genome-wide association studies data pertinent to T2D were sourced from the DIMANTE consortium and the FinnGen database. Techniques including inverse variance weighting (IVW), weighted mode, weighted median, and Mendelian randomization-Egger were employed for causal inference, complemented by sensitivity analysis. Genetically predicted higher phenylalanine (IVW: odds ratio = 1.10 95% confidence interval 1.04–1.17, *P* = 1.5 × 10^−3^, q_pval = 3.4 × 10^−2^) and dihomo-gamma-linolenic acid (IVW: odds ratio = 1.001 95% confidence interval 1.0006–1.003, *P* = 3.7 × 10^−3^, q_pval = 4.1 × 10^−2^) levels were directly associated with T2D risk. Conversely, no causal relationships between other nutrients and T2D were established. We hypothesize that phenylalanine and dihomo-gamma-linolenic acid contribute to the pathogenesis of T2D. Clinically, the use of foods with high phenylalanine content may pose potential risks for patients with a heightened risk of T2D. Our study provides evidence supporting a causal link between dietary nutrient intake and the development of T2D.

## 1. Introduction

Diabetes mellitus affects more than 35 million individuals in the United States. Projections indicate that by 2030, the prevalence of diabetes among Americans will rise to over 54 million, which could result in nearly 400,000 deaths each year.^[1]^ Type 2 diabetes (T2D) is a complex disease characterized by impaired β-cell function in the pancreas and insulin resistance in peripheral tissues, culminating in its clinical manifestation.^[2]^ Delays in treatment could lead to complications such as systemic macrovascular and microvascular disorders.^[3]^ Approximately 90% to 95% of diagnosed diabetes cases are type 2, a leading cause of renal failure, lower limb amputations, and adult blindness.^[4]^

The causation and etiology of T2D likely involve complex interactions among genetic, behavioral, and environmental factors. Contributing factors include a sedentary lifestyle, unhealthy diet, obesity, familial predisposition, aging, and even race or ethnicity.^[5]^ Modifiable risk factors are also associated with an increased risk of diabetes.^[6]^ Epidemiological research has shown that lower levels of vitamin E in plasma are associated with an increased risk of T2D.^[7]^ Research conducted by Hongbing Sun and colleagues suggests that a moderate dose of vitamin C supplementation, ranging from 500 to 1000 mg per day, may be beneficial for individuals with T2D.^[8]^ However, clinical studies by Sesso HD and colleagues have noted that vitamin C may not play a similar role in diabetes intervention.^[9]^ Metals including copper, iron, and zinc are strongly associated with T2D.^[10,11]^ A meta-analysis (involving 3978 participants) suggests that zinc supplementation can improve fasting blood glucose levels. Conversely, a clinical trial indicates no direct link between zinc supplementation and the progression of diabetes.^[12,13]^ Elevated serum amino acid levels are commonly observed in obese individuals, suggesting a potential role for amino acids in modulating insulin resistance.^[14]^ Recent studies, both cross-sectional and longitudinal, emphasize the critical role of amino acids in the progression of T2DM and in insulin resistance.^[15]^ and insulin resistance.^[16]^

Research has demonstrated that elevated levels of branched-chain amino acids (including leucine, valine, and isoleucine) lead to mTOR phosphorylation of insulin receptor substrate 1, disrupting insulin signal.^[17]^ Although some research indicates uncertainty about the adverse impact of elevated BCAA levels in diabetic patients, branched-chain amino acids, especially leucine, are recognized for their substantial role in anabolic metabolism, potentially enhancing insulin secretion from pancreatic β-cells.^[18,19]^ A systematic review and meta-analysis encompassing^[12]^ studies on n-3 polyunsaturated fatty acids and their impact on glycemic control in randomized controlled trials revealed no significant reduction in fasting insulin (fins), glycosylated hemoglobin^[20]^ However, some studies have reported that dietary supplementation with N-3 polyunsaturated fatty acids significantly lowers fasting blood glucose levels, HbA1c, and enhances insulin sensitivity.^[21,22]^ As indicated by the aforementioned research, there appears to be a correlation between dietary nutrients and T2D, potentially playing a role in the disease’s pathogenesis. However, most of this evidence originates from observational studies, which frequently exhibit inconsistencies and are subject to selection bias and unmeasured confounders. The causal relationship between dietary nutrients and T2D, and whether they increase risk or offer protection, remains unclear. Therefore, we have conducted a comprehensive study on this.

Mendelian randomization (MR) is a crucial tool in epidemiology, effectively utilizing genome-wide association studies (GWAS) data to analyze outcomes and employ genetic variations as instrumental variables (IVs) in exploring associations between relevant exposures and outcomes.^[23,24]^ Genetic variations remain unaffected by potential confounders, thus allowing for the avoidance of typical observational study limitations.^[25,26]^ In this study, we employed two-sample Mendelian randomization to investigate potential causal relationships between 4 categories of dietary nutrients and T2D.

## 2. Methods

### 2.1. Study design

This two-sample Mendelian randomization study utilizes aggregated autosomal genetic association data to evaluate the causal relationship between various dietary nutrients and T2D. Mendelian randomization operates on 3 core assumptions: correlation, independence, and exclusion restriction.^[27]^ The relevance assumption posits that genetic instruments, such as single nucleotide polymorphisms (SNPs), are employed to predict exposure. Independence suggests that the genetic instruments are free from confounding, and the confounding factors function independently of the exposure outcomes. Exclusion restriction denotes that the genetic instruments are independent of the outcomes of exposure, implying an absence of horizontal pleiotropy or selection bias.

### 2.2. Data sources

The primary outcome of the study was T2D, employing a meta-analysis of diabetes in European descendants from the DIAMANTE consortium for cross-ethnic association studies (cases: 80,154, controls: 853,816). Adjustments in the GWAS were made for age, sex, and study-specific covariates.^[28]^ Additionally, T2D summary statistics from FinnGen r9 (released on May 11, 2023) were selected for validation purposes (cases: 57,698, controls: 308,252). Initiated in 2017, the FinnGen study is a large-scale national cohort study that combines genetic data from Finnish biobanks with digital health record data from Finnish health registries. This integration represents a synergy of Finnish registry data and existing cohorts. Adjustments for genetic associations were made based on age, sex, principal components, and genotyping batches.^[29]^ Replication tests in this study utilized data from the FinnGen database as outcomes. Genetic associations were adjusted based on age, sex, principal components, and genotyping batches.

A search was conducted in the IEU database, PubMed, and the GWAS Catalog for published GWAS pertaining to individuals of European ancestry, with the latest search conducted in May 2023. A total of 29 potentially relevant micronutrients were identified, including branched-chain amino acids such as isoleucine, leucine, phenylalanine, and valine,^[30]^ lysine, methionine, and tryptophan^[31]^; unsaturated fatty acids such as arachidonic acid (AA), α-linolenic acid, docosahexaenoic acid (DHA), docosapentaenoic acid (DPA), dihomo-gamma-linolenic acid (DGLA), eicosapentaenoic acid (EPA), gamma-linolenic acid (GLA), and linoleic acid (LA)^[32,33]^ Metal elements identified include calcium (Ca), copper (Cu),^[34]^ iron (Fe),^[35]^ magnesium (Mg), selenium (Se),^[34]^ zinc (Zn).^[34]^ Vitamins identified include vitamin A,^[36]^ carotenoids,^[36]^ vitamin B6,^[36]^ vitamin B12,^[36]^ vitamin C,^[36]^ vitamin E,^[36]^ and 25-hydroxyvitamin D.^[37]^ The cohorts for exposure and outcome consisted exclusively of individuals with European ancestry, aimed at minimizing ethnic stratification bias.^[27]^ All data used in this study originated from research that had obtained appropriate subject consent and ethical endorsement, eliminating the need for additional ethical approval from an institutional review board. Detailed information regarding the GWAS datasets used in this study is available in Table 1.

**Table 1:** Genome-wide association studies data sources for exposure factors.

Exposure	Study or consortium	PMID or GWAS ID	Sample size	SNP	Ancestry
Isoleucine	Kettunen J et al^[30]^	27005778	24,776	12,076,452	European
Leucine	Kettunen J et al^[30]^	27005778	24,728	12,078,191	European
Phenylalanine	Kettunen et al^[30]^	27005778	22,663	12,042,964	European
Valine	Kettunen et al^[30]^	27005778	24,900	12,092,490	European
Lysine	Shin SY et al^[31]^	24816252	7812	2,545,686	European
Methionine	Shin SY et al^[31]^	24816252	7795	2,545,691	European
Tryptophan	Shin SY et al^[31]^	24816252	7804	2,545,641	European
Calcium	MRC-IEU^[36]^	ukb-b-8951	64,979	9,851,867	European
Copper	Evans DM et al^[34]^	23720494	2603	2,543,646	European
Selenium	Evans DM et al^[34]^	23720494	2874	2,451,527	European
Zinc	Evans DM et al^[34]^	23720494	2603	2,543,610	European
Iron	Benyamin B et al^[35]^	25352340	23,986	2,096,457	European
Magnesium	MRC-IEU^[36]^	ukb-b-7372	64,979	9,851,867	European
Vitamin A	MRC-IEU^[36]^	ukb-b-17406	62,991	9,851,867	European
Carotene	MRC-IEU^[36]^	ukb-b-16202	64,979	9,851,867	European
Vitamin B6	MRC-IEU^[36]^	ukb-b-7864	64,979	9,851,867	European
Vitamin B12	MRC-IEU^[36]^	ukb-b-19524	64,979	9,851,867	European
Vitamin C	MRC-IEU^[36]^	ukb-b-19390	64,979	9,851,867	European
25-hydroxyvitamin D	Revez JA et al^[37]^	32242144	496,946	6,896,093	European
Vitamin E	MRC-IEU^[36]^	ukb-b-6888	64,979	9,851,867	European
α-linolenic acid (ALA)	Lemaitre RN et al^[32]^	21829377	8866	2,775,163	European
Eicosapentaenoic acid (EPA)	Lemaitre RN et al^[32]^	21829377	8866	2,613,088	European
Docosapentaenoic acid (DPA)	Lemaitre RN et al^[32]^	21829377	8866	2,602,771	European
Docosahexaenoic acid (DHA)	Lemaitre RN et al^[32]^	21829377	8866	2,613,089	European
Diho-gamma-linolenic acid (DGLA)	Guan W et al^[33]^	24823311	8631	2,775,309	European
γlinolenic acid (GLA)	Guan W et al^[33]^	24823311	8631	2,775,164	European
Linoleic acid (LA)	Guan W et al^[33]^	24823311	8631	2,788,039*	European
Arachidonic acid (AA)	Guan W et al^[33]^	24823311	8631	2,788,039	European

ALA = α-linolenic acid, GWAS = genome-wide association studies, SNP = single nucleotide polymorphism.

### 2.3. Statistical analysis

Eligible SNPs were selected as IVs for the exposure. The selection criteria for IVs were: (1) significant association of SNPs with the exposure (*P* < 5 × 10^−6^) and absence of linkage disequilibrium (r^2^ = 0.01, window size = 10,000). (2) non-rare SNPs (MAF ≥ 0.01). SNPs absent in the GWAS results were identified through the online SNP NCBI database. SNPs lacking available proxies were excluded. The association strength between IVs and the exposure factor was assessed by calculating the F-statistic. The F-statistic was determined using the formula: F = R^2^ × (N − 2)/ (1 − R^2^).^[38]^ To reduce bias from weak instruments, SNPs with an F-statistic over 10 were selected for further analysis. Additionally, SNPs exhibiting allelic inconsistency and palindromic SNPs with mismatched MAF were excluded in the harmonization process. The Steiger test was applied to all SNPs to mitigate the risk of reverse causality.^[39]^ Tables (Table S1, Supplemental Digital Content, http://links.lww.com/MD/M426 and Table S2, Supplemental Digital Content, http://links.lww.com/MD/M427) provide detailed information on the genetic IVs used. Following screening, methionine, lysine, tryptophan, GLA, and α-linolenic acid were excluded due to the unavailability of SNPs.

### 2.4. MR analysis

The inverse-variance weighted (IVW) method serves as the primary approach for causality analysis in this study; it integrates SNP results on SNP exposure effects via weighted linear regression and accommodates over-dispersion.^[40]^ The multiplicative random-effects model of IVW was chosen for analysis, as it offers an average of the actual estimates. To enhance result robustness, additional sensitivity analyses were employed, including the weighted mode method, the weighted median method, and the MR-Egger method.^[41]^ weighted median method, and the MR-Egger method. The weighted median method produces the median of the weighted empirical density function of the ratio estimates.^[42]^ This method offers more effective estimates compared to the simple median method, especially when at least 50% of the IVs are valid, and it remains sensitive to the addition or removal of IVs.^[43]^ The MR-Egger method facilitates obtaining more accurate estimates, even in the presence of SNP pleiotropy.^[44]^ The IVW method is the key to ascertaining the impact of exposure on outcomes.^[45]^ When only 1 SNP is available, the Wald ratio method is employed to determine the impact of a single IV on T2D.^[23]^ Furthermore, through the application of leave-one-out sensitivity analysis, it was assessed whether individual influential SNPs exerted an impact on the results. Cochrane Q heterogeneity test was used to evaluate the degree of heterogeneity, with *P* < .05 indicating a high rate of heterogeneity. For cases with high heterogeneity (*P* < .05), the multiplicative random-effects IVW method is utilized.^[46]^ The MR-Egger method identifies potential pleiotropy by checking if the intercept between exposure and outcome is zero.^[23]^ Once adjusted for pleiotropy, this method yields a more conservative estimate of the causal effect.^[47]^ MR-PRESSO^[48]^ was utilized to detect extensive horizontal pleiotropy in all outcomes, thereby aiming to minimize bias. RadialMR imaging was employed for the automatic detection of outliers.^[49]^ The Benjamini–Hochberg correction, controlling the false discovery rate (FDR), was applied for multiple testing correction in T2D, with associations having a Benjamini–Hochberg adjusted *P* < .05 considered significant.^[50]^ Funnel plots were utilized to illustrate the bias in results. The comprehensive data analysis of this study was conducted using R software (version 4.2.2). The R packages employed included TwoSampleMR, MR-PRESSO, RadialMR, and the Stats package.

## 3. Results

### 3.1. The causal role of nutrients in T2D

**Figure 1.**
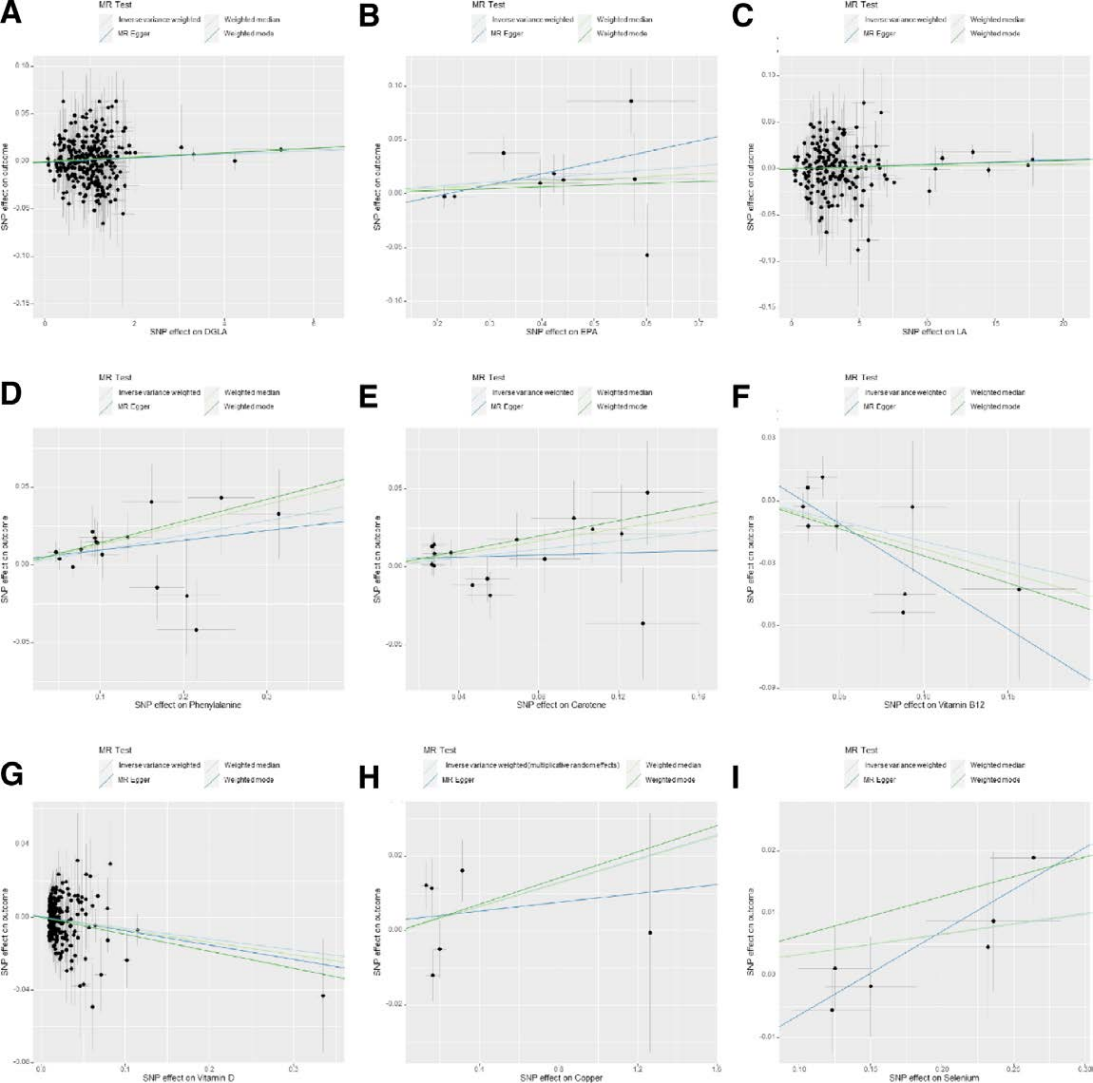
Scatter plot of genetic causality between Nutrients and T2D using different MR methods in DIAMANTE. (A) DGLA, (B) EPA, (C) linoleic acid, (D) phenylalanine, (E) carotene, (F) vitamin B12, (G) vitamin D, (H) copper, (I) selenium. DGLA = diho-gamma-linolenic acid, EPA = eicosapentaenoic acid.

**Figure 2.**
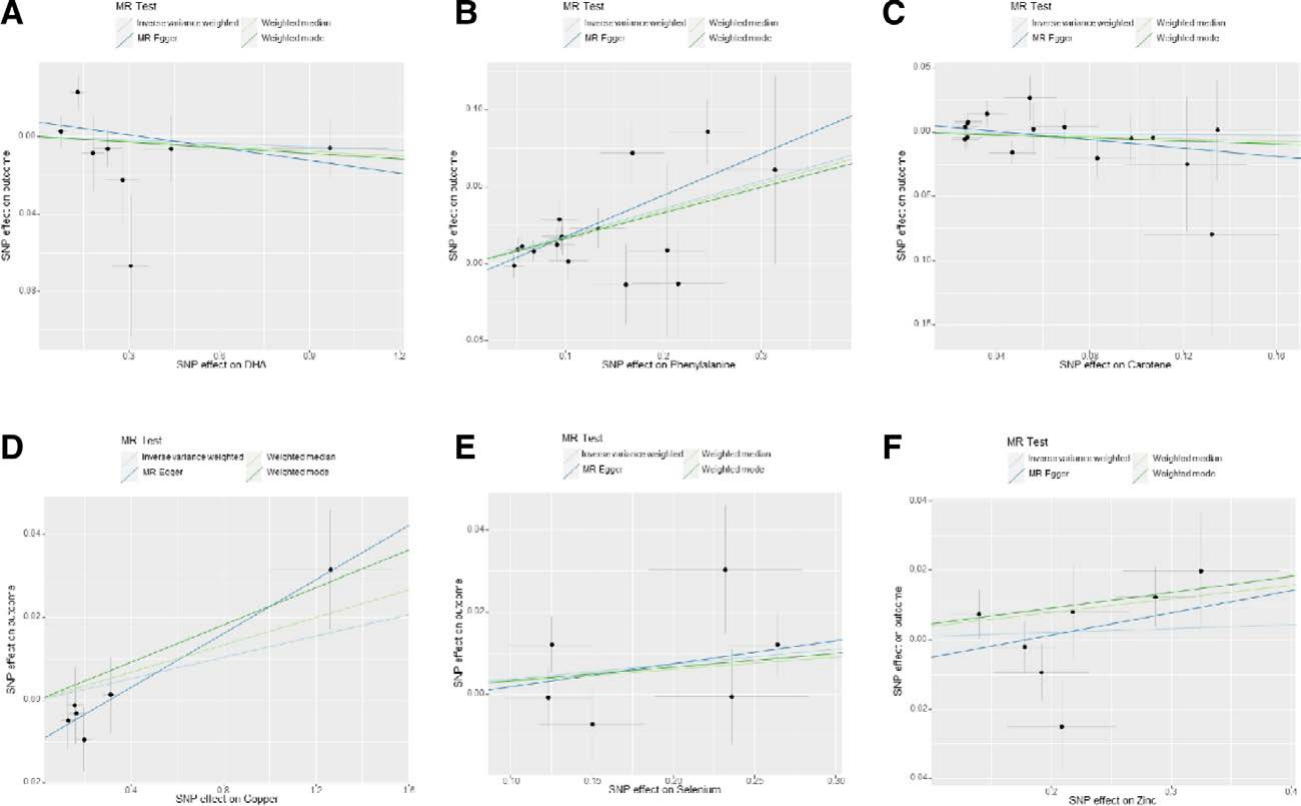
Scatter plot of genetic causality between Nutrients and T2D using different MR methods in FinnGen. (A) DHA, (B) phenylalanine, (C) carotene, (D) copper, (E) selenium, (F) zinc. DHA = docosahexaenoic acid, T2D = type 2 diabetes.

**Figure 3.**
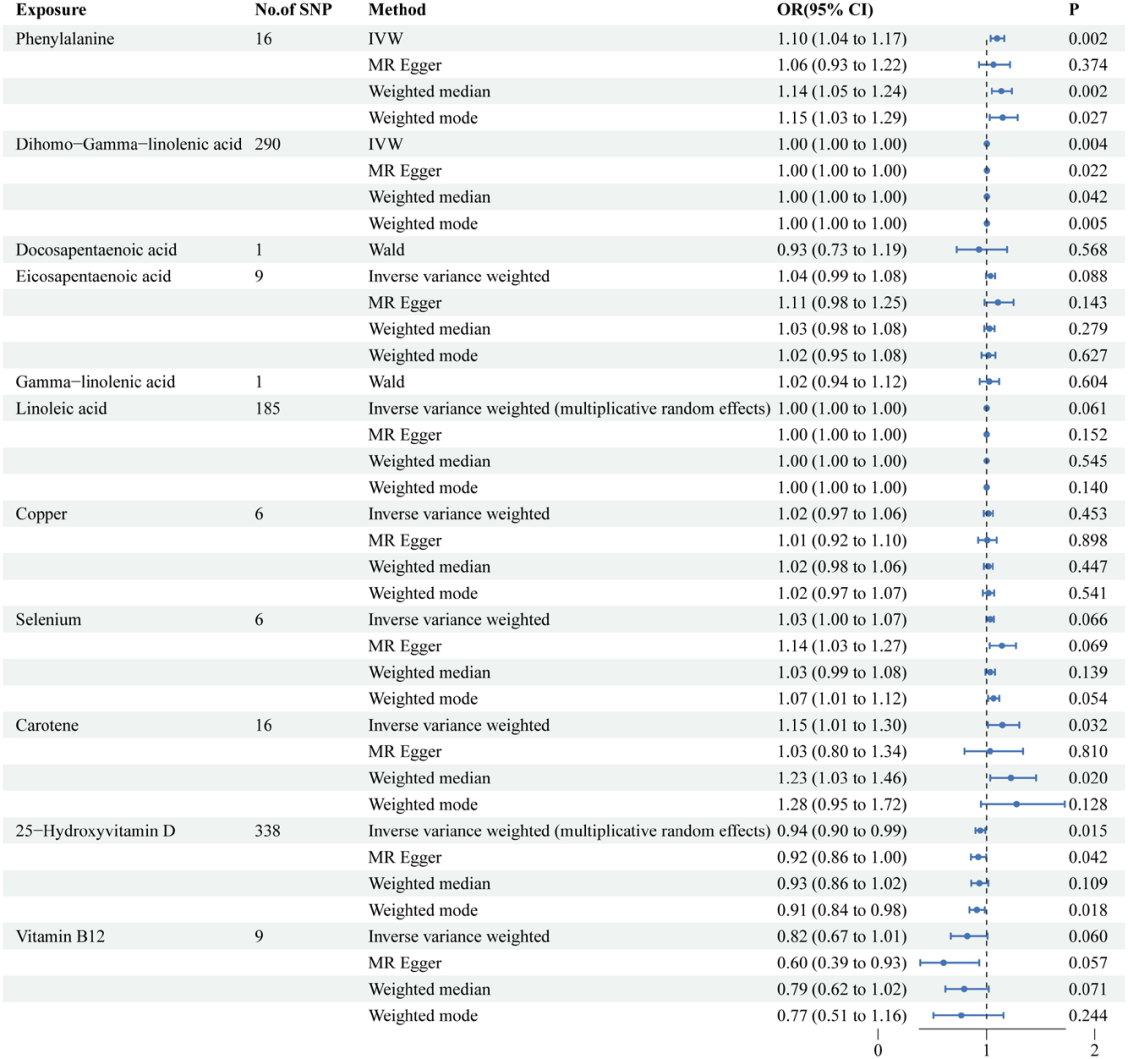
Associations of genetically predicted nutrients with risk of T2D in DIAMANTE. CI = confidence interval, OR = odds ratio, T2D = type 2 diabetes.

**Figure 4.**
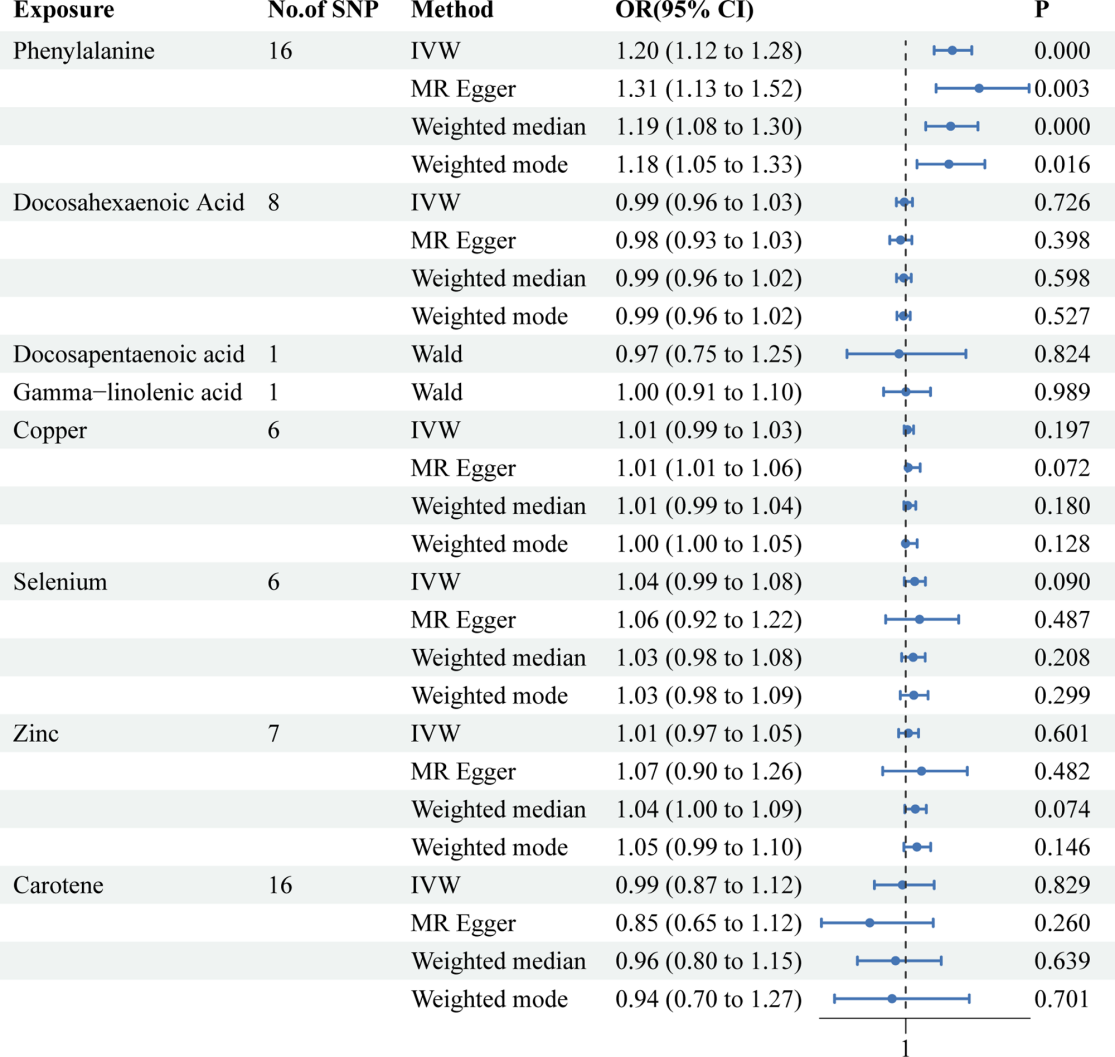
Associations of genetically predicted nutrients with risk of T2D in FinnGen. CI = confidence interval, OR = odds ratio, T2D = type 2 diabetes.

After selecting SNPs based on F-statistics and assessing them using the Steiger test to reduce potential reverse causality risks, a subsequent correction of *P*-values was carried out. The DIAMANTE analysis revealed that both phenylalanine (IVW: odds ratio [OR] = 1.10 95% confidence interval (CI) 1.04–1.17, *P* = 1.5 × 10^−3^, q_pval = 3.4 × 10^−2^) and DGLA (IVW: OR = 1.001 95% CI 1.0006–1.003, *P* = 3.7 × 10^−3^, q_pval = 4.1 × 10^−2^) are causally related to T2D. However, DPA, EPA, gamma-linolenic acid, AA, linoleic acid, copper, selenium, vitamin B12, vitamin D, and beta-carotene were found to have no causal relationship with T2D. Results for other nutrients varied across different MR analytical methods and thus were not deemed conclusive. For DPA and gamma-linolenic acid, where only a single SNP met the F-statistic threshold, Wald analysis was conducted, yielding negative results with limited research significance. Notably, in the FinnGen study, genetic prediction results indicated an association between phenylalanine levels and T2D via IVW analysis (IVW: OR = 1.20; 95% CI 1.19–1.28; *p*, 1.14 × 10^−7^; *q_pval *= 2.50 × 10^−6^). In the analysis of the FinnGen database, DHA, DPA, GLA, copper, selenium, zinc, vitamin D, and carotenoids were found to have no causal relationship with T2D. (Fig. 1 scatter plot of DIAMANTE, Fig. 2 scatter plot of FinnGen, Fig. 3 forest plot of DIAMANTE, Fig. 4 forest plot of FinnGen, Table S3, Supplemental Digital Content, http://links.lww.com/MD/M428. The table offers a comprehensive explanation of the outcomes of Mendelian randomization.)

### 3.2. Sensitivity analysis

In the FinnGen study, Cochrane Q test indicated that the IVW method demonstrated no heterogeneity across studies (phenylalanine Q statistic = 17.85;*P* = .27; DHA Q statistic = 12.23;*P* = .093; copper Q-statistic = 5.34;*P* = .38; selenium Q-statistic = 6.39;*P* = .27 zinc Q-statistic = 9.59; *P *= .14; carotene statistic = 13.95; *P *= .53). Similarly, in the DIAMANTE analysis, no evidence of heterogeneity was observed (DGLA Q-statistic = 239.66; *P *= 9.8 × 10^−1^; EPA Q-statistic = 12.22; *P *= .14; LA Q-statistic = 158.47; *P *= 9.1 × 10^−1^; copper Q-statistic = 11.38; *P *= 4.4 × 10^−2^; selenium Q-statistic = 5.14; *P *= 4.0 × 10^−1^; vitamin B12 Q statistic = 12.11; *P *= 1.5 × 10^−1^; vitamin D = 236.22; *P *= 7.7 × 10^−2^; carotene Q-statistic = 15.80; *P *= 3.9 × 10^−1^; phenylalanine Q-statistic = 12.35; *P *= 6.5 × 10^−1^).

Considering that the MR-Egger intercepts for the majority of MR analyses (except for AA in the DIAMANTE study) center around zero, these intercepts indicate no evidence of unbalanced pleiotropy (P_intercept > 0.05). For reliable results, outliers detected by MR-PRESSO and RadialMR were eliminated (Table S4, Supplemental Digital Content, http://links.lww.com/MD/M429, Fig. S1, Supplemental Digital Content, http://links.lww.com/MD/M430, Fig. S2, Supplemental Digital Content, http://links.lww.com/MD/M431. The table and figure depict the outcomes of sensitivity analysis.).

Following multiple testing corrections, a statistically significant causal relationship between phenylalanine and T2D was established. In DIAMANTE and FinnGen studies, with T2D as the outcome, a significant association between phenylalanine and the risk of T2D was observed (IVW: OR = 1.10 95%CI 1.04–1.17, *P* = 1.5 × 10^−3^, q_pval = 3.4 × 10^−2^) and (IVW: OR = 1.20; 95%CI 1.19–1.28; *P* = 1.14 × 10^*−*7^; q_pval = 2.50 × 10^−6^). Results from the sensitivity analyses indicated that there is minimal evidence suggesting a link between the circulating concentrations of other nutrients and the risk of T2D.

## 4. Discussion

In this MR analysis, we provided evidence that genetically predicted higher phenylalanine and DGLA may potentially increase the risk of T2D. In the DIAMANTE study, the impact of DGLA was not found to be significant, and the FinnGen database results do not suggest that DGLA increases the risk of T2D. These differing conclusions may stem from variations in selection criteria, case definitions, or potentially different genetic structures.^[51]^ Apart from phenylalanine and DGLA, this study found no causal relationship between other nutrients and T2D.

As T2D research advances, metabolites such as amino acids and fatty acids are increasingly being recognized as markers for the disease in clinical studies. Currently, the majority of literature regarding the link between phenylalanine and T2D suggests an association with the disease’s risk. In a controlled study conducted by Miguel Ruiz-Canela et al, dietary interventions in patients led to the measurement of amino acids in plasma, revealing a notable correlation between phenylalanine levels and increased risk of T2D, alongside a significant linear dose-response pattern.^[52]^ Furthermore, a systematic review has demonstrated a positive correlation between phenylalanine and the risk of T2D.^[53]^

The potential mechanisms behind this association may involve phenylalanine’s role in stimulating insulin secretion from pancreatic β cells,^[54–57]^ and its potential exacerbation of T2D through hyperinsulinemia, leading to pancreatic β cell exhaustion.^[58]^ Research by Zhou Q et al demonstrated that phenylalanine modifies the insulin receptor β (IRβ), resulting in the inactivation of insulin signal transduction and glucose uptake. Mice fed diets high in phenylalanine or aspartame, a phenylalanine source, exhibited signs of insulin resistance and T2D symptoms.^[59]^

The role of DGLA in T2D risk has been noted previously. Research has shown a correlation between increased serum DGLA levels in Japanese T2D patients and factors like obesity, body fat accumulation, high ALT levels, and insulin resistance.^[60]^ Furthermore, a study by Ju-Sheng Zheng and colleagues highlighted an increased risk of T2D associated with elevated serum DGLA concentrations.^[61]^ However, currently, no research on the molecular mechanisms behind these findings is available.

Other nutrients have been linked to T2D in various observational studies, this study did not establish a causal relationship with T2D. Mendelian randomization research by Anna-Maria Lampousi and others also concluded that β-carotene, vitamins C and E, selenium, and zinc are not causally linked to T2D, aligning with our conclusions.^[62]^ Studies by Hao Liang et al have suggested that higher linoleic acid levels might reduce the risk of T2D, yet our analysis using the FinnGen and DIAMANTE databases did not corroborate this finding.^[63]^ Research by Benjamin De La Barrera and Despoina Manousaki indicated that vitamin D does not influence the risk of T2D in adolescents across different ethnicities, consistent with our study’s results.^[64]^

This MR study has the following advantages: Firstly, this research utilizes publicly available GWAS data for causal inference, studying dietary factors that may influence T2D onset using MR to mitigate confounding factors or reverse causation, common issues in observational studies.^[65–67]^ Secondly, in addition to the primary IVW method, auxiliary methods such as MR-Egger and the Wald ratio were also utilized. Furthermore, a variety of sensitivity analysis methods were employed to validate the results. Overall, this MR study offers significant insights into the causal relationship between diet-related nutrients and the risk of developing T2D, as well as glycemic traits.

However, this study has limitations, including that all analyses were conducted solely with European participants, making generalization to other populations difficult. Additionally, the study lacked comprehensive sensitivity analysis to assess the possibility of horizontal pleiotropy, although the MR-Egger intercept test indicated no clear evidence of such pleiotropy. Thirdly, some heterogeneity was observed in the results. Nevertheless, the random effects IVW method remained the primary analytical approach, effectively controlling for heterogeneity in the pooled data. Fourthly, reliance solely on genetic-level evidence limited further observational studies and mediation analyses to verify specific regulatory mechanisms of the causal relationship between dietary nutrients and T2D. Fifthly, the study focused solely on linear causal relationships, treating nutrient levels as continuous variables. Therefore, future research should encompass a broader and more diverse population across different ancestries and cultures and conduct nonlinear MR analysis to explore the potential nonlinear effects of dietary nutrient levels on T2D.

## 5. Conclusion

In summary, this MR study observed that genetically predicted levels of phenylalanine and DGLA are positively associated with the risk of developing T2D. Although the mediating processes involved require further elucidation, this insight proves valuable in understanding the relationship between dietary nutrients and T2D. Further research focusing on the monitoring of phenylalanine and DGLA levels as long-term indicators is recommended. To diminish the incidence of T2D, future efforts should concentrate more on elucidating the potential links between nutrients and T2D.

## Author contributions

**Conceptualization:** Dong Guo.

**Data curation:** Ruixiang Kang, Jiawei Wang.

**Formal analysis:** Ruixiang Kang, Jiawei Wang.

**Writing – original draft:** Ruixiang Kang.

**Writing – review & editing:** Zhencong Xie.

## Supplementary Material












