# Supplementary material for: Association of dietary nutrient intake with type 2 diabetes: A Mendelian randomization study
Source: Medicine (Baltimore). 2024 May 10;103(19):e38090. doi: 10.1097/MD.0000000000038090 (PMC11081547; doi:10.1097/MD.0000000000038090)
Supplement: Supplementary file 5 [file medi-103-e38090-s005.docx]

Supplementary Figure 1


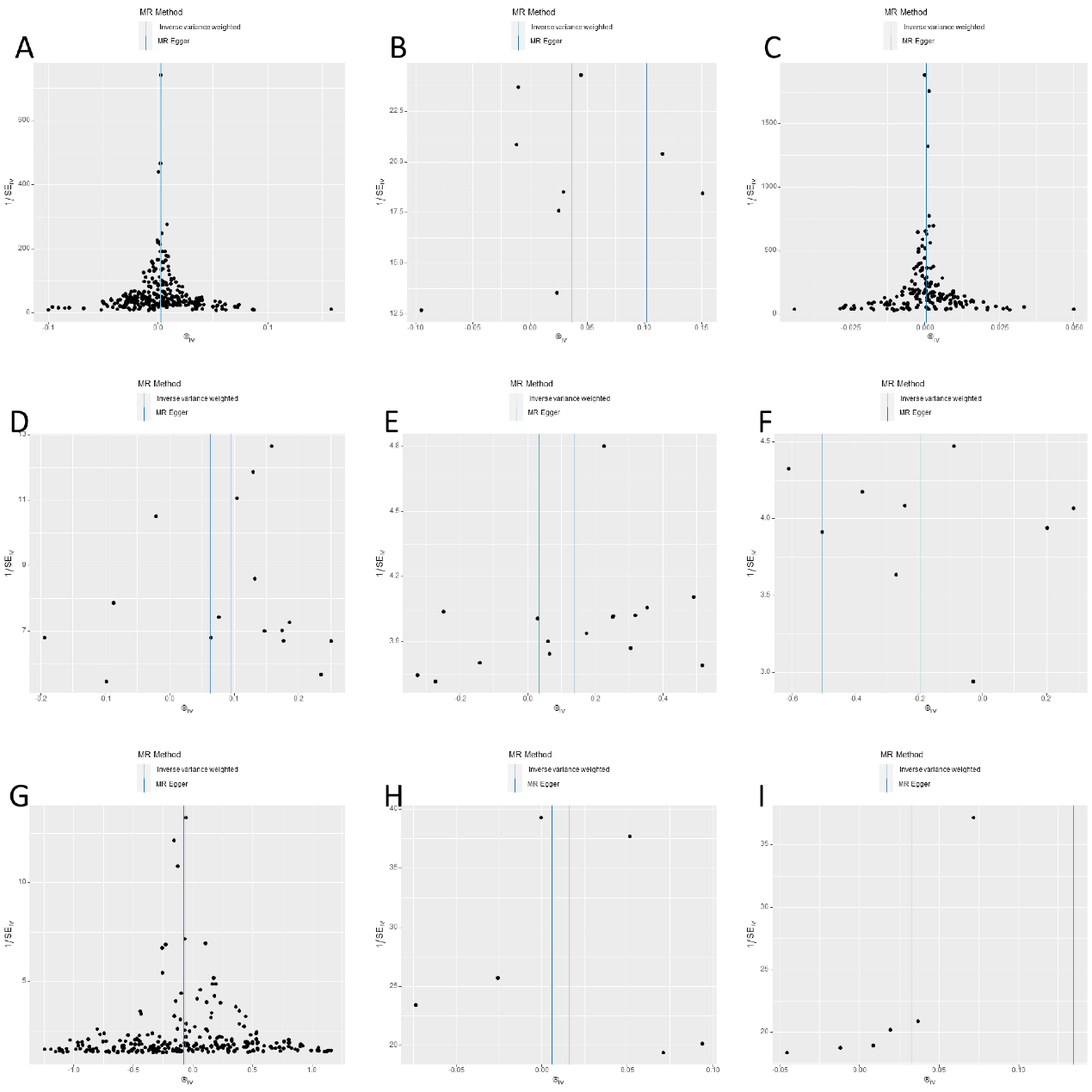


Supplementary Figure 1-The funnel chart depict the outcomes of sensitivity analysis in DIAMANTE. (A) DGLA (B) EPA (C) linoleic acid (D) phenylalanine (E) carotene (F) vitamin B12 (G) vitamin D (H) copper (I) selenium
